# Nitric Oxide Synthase 2 Polymorphisms (rs2779248T/C and rs1137933C/T) and the Risk of Type 2 Diabetes in Zahedan, Southeastern Iran

**Published:** 2018-11

**Authors:** Yasaman GARME, Mahdiyeh MOUDI, Ramin SARAVANI, Hamidreza GALAVI

**Affiliations:** 1. Cellular and Molecular Research Center, Zahedan University of Medical Sciences, Zahedan, Iran; 2. Dept. of Clinical Biochemistry, School of Medicine, Zahedan University of Medical Sciences, Zahedan, Iran

**Keywords:** Type 2 diabetes mellitus, Single nucleotide polymorphism, Nitric oxide synthase type II, Inducible nitric oxide synthase

## Abstract

**Background:**

Nitric oxide (NO) has been associated with insulin resistance and type 2 diabetes (T2D). NO is synthesized enzymatically from l-arginine (l-Arg) by three NO synthase (NOS) isoforms, Neuronal NOS (nNOS or NOS1), Inducible NOS (iNOS or NOS2), and Endothelial NOS (eNOS or NOS3). The impact of NOS2 gene polymorphism was investigated on the susceptibility of T2D in a sample of Iranian population (Southeastern of Iran).

**Methods:**

In 2015, the present case-control study was conducted on 152 T2D patients and 157 healthy control subjects (HCs) referring to Bu-ali Hospital of Zahedan, eastern Iran. Genotyping of NOS2 rs2779248T/C and rs1137933C/T variants were done using the Tetra-Amplification Refractory Mutation System Polymerase Chain Reaction (Tetra-ARMS PCR) method.

**Results:**

CT genotype of rs1137933C/T was significantly associated with increased risk of T2D (*P*<0.0001). The T allele of this single nucleotide polymorphism (SNP) was also strongly associated with T2D risk (*P*<0.0001). For rs2779248 T/C, TC genotype of this SNP decreased the risk of T2D (OR=0.25 95%CI= 0.15–0.42, *P*<0.0001); however, CC genotype of this SNP increased the risk of T2D (*P*<0.005). There was no significant association between clinical-demographic characteristics of T2D group with respect to both SNPS in dominant.

**Conclusion:**

CT genotype and C allele of NOS2 rs1137933 C/T polymorphism were associated with a higher risk of T2D, and no association was observed between T allele of NOS2 rs2779248 T/C polymorphism and T2D while TC genotype of this SNP decreased the risk of T2D in the study participants.

## Introduction

An important public health issue in world is diabetes mellitus characterized by increased blood glucose level (hyperglycemia) and associated with some high-risk behaviors such as smoking, associated with many diabetes complications such as nephropathy (1–3). Lack of production of enough insulin by pancreas or responding correctly to insulin production by body cells are two main causes of diabetes (4). Three main types of diabetes mellitus (DM) has been defined: Type 1 DM, Type 2 DM, and gestational diabetes (5, 6). There were about 415 million people with diabetes worldwide in 2015, 90% of whom had type 2 DM. This rate was equal in men and women. In 2014, the cause of 4.9 million deaths in the world was diabetes, declared by International Diabetes Federation (IDF). A lot of them die because of cardiovascular diseases (7, 8). The most common type of diabetes mellitus is Type 2 diabetes (T2D), and lifestyle factors including obesity, lack of physical activity, poor diet, stress, urbanization, and genetic factors contribute to this type of diabetes (9, 10).

Diabetes has genetic heterogeneity (4, 11, 12). Some genes are associated with T2D, expressed in beta cells or involved in insulin secretion pathways, and their associations are confirmed by systematic review and meta-analysis studies. Moreover, genetic factors play key roles in the pathogenesis and progression of T2D (13–18).

One of these genes is called nitric oxide synthase (NOS). NOSs are a group of enzymes which catalyze the manufacturing of nitric oxide from L-arginine and this enzyme is necessary for cellular signaling. Moreover, insulin secretion is regulated by NOS. Each of NOS family members is encoded by different genes. Constitutive (cNOS) and inducible (iNOS) are two isoforms of NOS (19). iNOs gene is located on chromosome 17q11. This gene has 27 exons; its transcription start site is in exon 2, and its stop codon is in exon 27 (20).

Some polymorphisms of nitric oxide synthase genes have been involved in several diseases in different populations (21–24). Moreover, they indicated some polymorphisms in the coding region of the iNOS gene may alter the product activity while some polymorphisms in the promoter region may change the level of gene product (25). Considering the biological and pathologic significance of NOS2, genetic polymorphisms of this gene are associated with inflammatory disease even T2D (25–28). Genetic variations in this gene may contribute to the occurrence and development of T2D.

The present investigation set to evaluate the association of NOS2 rs2779248T/C and NOS2 rs1137933C/T SNPs with susceptibility to T2D. The reason for selection of these two SNPs is that many inflammatory diseases are associated with these polymorphisms such as T2D in previous study (28, 29). Therefore, genotyping analyses were conducted for two SNPs in 152 T2DM cases and another 157 healthy controls in Southeastern of Iranian population.

## Materials and Methods

The study was approved by the Ethics Committee of the “Zahedan University of Medical Sciences” (ethical code: IR. ZAUMS. REC. 1394.44). Written informed consents were obtained from all participants.

In 2015, a total number of 309 individuals were included in this typical case-control study. Overall, 152 patient volunteers with T2D referring to Bu-ali Hospital of Zahedan, eastern Iran were included in the study group. The control group consisted of 157 healthy non-diabetic volunteers. For both groups, fasting blood sugar (FBS) level and/or a 2-h glucose level and HbA1C were analyzed. The inclusion criteria in T2D group were as follows: FBS more than 126 mg/dl and/or 2-h glucose>200, and HbA1C>6.7, and the inclusion criteria for healthy control were normal FBS and HgA1C (30, 31), and both groups were confirmed by at least two endocrinologists. The healthy group individuals should not have had any specific systemic diseases and any family relationship with the T2D group (exclusion criteria).

DNA samples were obtained from 3–5 cc peripheral blood, using salting out method (32). Geno-typing was based on Tetra ARMS PCR technique. For specific DNA amplification, 2 μL (∼80–100 ng) of genomic purified DNA was amplified in a volume of 20 μL reaction mixture containing 10 μL of PCR master mix, ready-to-use solution containing Taq DNA polymerase, deoxynucleotide triphosphates, MgCl2, reaction buffers (0.2 units/μL ampliqonTaq DNA polymerase, ampliqonTaq 2x master mix, Denmark), 4 μL free nuclease water, and 1 μL of each primer. The amplification products were submitted to thermocycler and were analyzed by electro-phoresis agarose gel allowing detection via ethidium bromide staining the corresponding geno-types. Specific thermocycling conditions and resulting DNA fragments are presented in Table 1. Statistical analysis was carried out using SPSS 16.0 (Chicago, IL, USA); demographic and clinical data were compared using the Pearson’s chi-square test (*χ*^2^) and the phi coefficient. The distribution of genotype and allele frequency of each NOS2 rs2779248T/C and rs1137933C/T polymorphisms were compared between different groups by the Fisher’s exact test, followed by comparative analysis based on dominant and recessive models.

**Table 1:** PCR primers sequences and condition of amplification

***Primer 5′-3′***	***Product***	***Method and condition(Temperature/Time)***
Rs1137933C/T		Tetra-ARMS
Fo: TGACAGATACGAGTGGTTTCGGGAACTG	C allele: 219	95 °C/5min,
Ro: TGATCTCAACGACAGCCTAGTCTTTCCA	T allele: 183	95 °C/1min,
FI: CAGAGATCGGAGTCCGGGACTTCTGTTAC	two outer primers: 345	62 °C/1min,
RI TACCTACAGGATGTTGTAACGCTGGCCA		72 °C/1min and 72 °C/5 min
Rs2779248T/C		Tetra-ARMS
Fo: GTGGGTGACCTGATCTTGCTGTTACATC	C allele: 198	95 °C/5min,
Ro: TTCCATACTGTCAATATTCCCCCAGCTT	T allele: 140	95 °C/1min,
FI: GTTCATCAGTAGGGTGGCTGCTAATAC	two outer primers: 284	57 °C/1min,
RI: ATAAAACCTGACTCCGTGGTGCCTATA		72 °C/1min and 72 °C/5 min

Odds ratio (OR) was calculated at 95% confidence interval (CI) to estimate the relative risk and strength of association. A *P*-value of 0.05 was considered as statistically significant. The Hardy–Weinberg equilibrium (HWE) was tested using χ^2^ for each SNP.

## Results

The study consisted of 152 T2D patients (41 males and 111 females, aged 54.25±9.71 yr) and 157 healthy control subjects (49 males and 108 females, aged 49.36±10.15). As far as gender and age are concerned, no significant difference was observed between the groups (*P*=0.453 and *P*=0.733, respectively). The clinical-demographic characteristics of T2D patients and healthy control (HC) subjects are shown in Table 2.

**Table 2:** Clinical-demographic characteristics of T2D patients and controls

***Variable***	***T2D(n=152) (n ± SD)***	***Controls(n=157) (n ± SD)***	***P-value***
Age(yr)	54.25±9.71	49.36±10.15	0.733
Sex(Female/male)	111/41	108/49	0.453
FBS(mg/dl)	169.00±76.61	96.65±16.61	<0.0001
TC(mg/dl)	176.91±41.51	184.05±34.11	0.018
TG(mg/dl)	146.40±79.14	156.25±97.99	0.127
HDL(mg/dl)	57.34±19.22	53.13±15.89	0.028
LDL(mg/dl)	90.30±30.14	105.28±28.85	0.860

FBS: Fast blood sugar, TC: Total Cholesterol, TG: Triglyceride, HDL; High-density lipoprotein, LDL: Low-density lipoprotein

In terms of FBS, total cholesterol (TC), and high-density lipoprotein (HDL), there were significant differences between T2D and HC (*P*<0.0001, *P*< 0.018, and *P*=0.028, respectively). Genotypes and allele frequencies of NOS2 polymorphism are provided in Table 3. rs1137933 C>T genotype significantly increased the risk of T2D in co-dominant (*P*<0.0001, CT vs. CC).

**Table 3:** Genotypic and allelic frequencies of NOS2 polymorphisms in T2D patients and control subjects

***NOS2***	***Type 2 Diabetes***	***Control***	***OR(95%CI)***	***P-value***
***Polymorphisms***	***n(%)***	***n(%)***		
rs1137933C/T				
CC	135(88.8%)	157(100%)	1.00	-
CT	16(10.5%)	0	-	<0.0001
TT	1(0.7%)	0	-	0.464
Allele				
C	286(94%)	314(100%)	1.00	-
T	18(6%)	0	-	<0.0001
Dominant				
CC	135(88.8%)	157(100%)	1.00	-
CT+TT	17(11.2%)	0(0%)	-	<0.0001
Recessive				
CC+CT	151(99.3%)	157(100%)	1.00	-
TT	1(0.7%)	0(0%)	-	0.23
rs2779248T/C				
TT	70(46.1%)	33(21%)	1.00	-
TC	66(43.4%)	124(79%)	0.25(0.15–0.42)	<0.0001
CC	16(10.5%)	0	-	0.005
Allele				
T	206(67.7%)	190(60.5%)	1.00	-
C	98(32.3%)	124(39.5%)	0.73(0.52–1.02)	0.065
Dominant				
TT	70(46%)	33(21%)	1.00	-
TC+CC	82(54%)	124(79%)	0.31(0.19–0.51)	<0.0001
Recessive				
TT+TC	136(89.5%)	157(100%)	1.00	-
CC	16(10.5%)	0(0%)	-	<0.0001

CI, confidence interval; OR, odds ratio

Furthermore, the T allele significantly rose the risk of T2D (*P*<0.0001) compared to C allele.

Moreover, SNP was associated with T2D in the dominant (*P*<0.0001, CC vs. CT+TT), but in the recessive, there was not such as relation (*P*=0.23). For rs2779248 T>C, TC vs. TT genotypes decreased the risk of T2D (OR=0.25 95%CI=0.15–0.42, *P*<0.0001). In contrast, the CC genotype was significantly associated with T2D as risk factor in comparison with the reference genotype (TT) (*P*<0.0001) although the C allele of this SNP did not show any association with T2D, either protective or risk compared to T allele. However, either the dominant or recessive was associated with T2D (OR=0.31 95%CI=0.19–0.51, *P*<0.0001 and *P*<0.0001, respectively).

The current study revealed on association between NOS2 polymorphisms in the dominant, and clinical-demographic characteristics of T2D patients are given in Table 4. The results represented no statistically significant association between these features and both SNPs, rs1137933C/T and rs2779248T/C in the dominant (CC vs. CT+TT and TT vs. TC + CC, respectively).

**Table 4:** Association between NOS2 polymorphisms with clinical-demographic characteristics of T2D patients

***Genotype***	***Age(yr)***	***Sex(Male/Female)***	***FBS(mg/dl)***	***TC(mg/dl)***	***TG(mg/dl)***	***HDL(mg/dl)***	***LDL(mg/dl)***
rs1137933C/T							
CC	54.18±10.04	38(M)/96(F)	169.37±78.81	176.25±40.08	147.56±80.42	56.78±18.21	90.01±30.85
CT+TT	54.82±6.74	3(M)/15(F)	166.06±58.09	181.76±52.09	137.76±70.51	60.68±24.85	92.05±26.2
*P*-value	0.082	0.563	0.221	0.148	0.248	0.056	0.870
rs2779248T/C							
TT	53.82±9.32	19(M)/51(F)	155.05±62.92	180.92±41.11	145.85±72.89	59.16±17.48	89.69±27.77
TC+CC	54.62±10.08	22(M)/60(F)	180.63±85.03	173.56±41.81	146.86±84.39	55.97±20.44	90.75±32.32
*P*-value	0.585	0.999	0.101	0.848	0.788	0.431	0.326

FBS: Fast blood sugar, TC: Total Cholesterol, TG: Triglyceride, HDL; High-density lipoprotein, LDL: Low-density lipoprotein

All the loci were done to the Hardy-Weinberg equilibrium (HWE) in both T2D and HC and in the whole population. In the T2D group, the genotype distribution of both SNPs rs1137933C/T and rs2779248T/C were in HWE (**χ**^2^= 0.46, *P*=0.496 and **χ**^2^ =0.01, *P*=0.939, respectively), while the genotype distribution of rs2779248T/C in the control was not in HWE (**χ**^2^=66.87, *P*<0.0001), and rs1137933C/T was not polymorphic in the study population and the whole population, for rs1137933C/T was in HWE (**χ**^2^= 2.2, *P*=0.137) and rs2779248T/C was not in HWE (**χ**^2^= 34.81, *P*<0.0001).

## Discussion

In this case-control study of T2D and HC, the association of NOS2 rs1137933C/T (Exon 10 (33)) and rs2779248T/C (Promoter (34)) SNPs with T2D risk was investigated. The relationship between polymorphism and susceptibility to T2D has not been evaluated in this population so far, thus this is the first study to demonstrate such an association. NOS2 or inducible NOS (iNOS) is one of three isoforms of NOS located on 17q11.2–q12 with 27 exons (35). NOS2 is associated with many inflammatory diseases such as rheumatoid (26), inflammatory bowel disease (27), and coronary heart disease (CHD) (36, 37), besides inflammatory mediators and stimulators inducing NOS2 (37). This study was designed on the basis of T2D as an inflammatory disease (38) and NOS2’s association with inflammation (e.g. CHD is a T2D complication).

The NOS2 rs1137933C/T and rs2779248T/C gene polymorphisms were significantly associated with T2D risk. The T-allele and CT genotype of NOS2 rs1137933C/T and CC genotype of NOS2 rs2779248T/C were significantly associated with increased risk of T2D, and TC genotype of NOS2 rs277 was significantly different between the case and control groups and had protective role for T2D. However, there was no significant difference regarding the TT genotype of NOS2 rs1137933C/T and C allele of the NOS2 rs2779248T/C between T2D and HC. There was no significant association between the clinical-demographic characteristics and both SNPs in the dominant.

There are a few data regarding the role of NOS2 polymorphism and T2D. In a case-control study, rs1137933C/T and/or rs2779248T/C were not significantly associated with T2D susceptibility (28). The current study, however, showed a significant association between rs1137933C/T and rs2779248T/C NOS2 regarding the risk of T2D. Moreover, rs1137933C/T was not associated with invasive pneumococcal disease (36), but this SNP was associated with vary early onset (VEO) inflammatory bowel disease (IBD) and VEO-ulcerative colitis in a case-control study in Spain (27). Nevertheless, in a cohort study, rs1137933C/T was not associated with VEOIBD (39). In the present study, the association between some experimental parameters and polymorphisms of NOS2 in the dominant was evaluated. In another study, both variants of NOS2 (rs1137933C/T and rs2779248T/C) were not associated with positive history of hyperlipidemia, but lipid profile separately was not analyzed (28). In contrast, to the current study the authors evaluated the association between lipid profile and gene polymorphisms in the dominant, revealing that age, gender, FBS, and lipid profile were not different with respect to rs1137933C/T NOS2 and rs2779248T/C NOS2 in the dominant.

Our study had some limitations. First, based on the published and Medline data several NOS2 polymorphisms have been detected in humans, while we investigated just two polymorphisms. Second, relatively small sample size; however Calorie intake, sedentary lifestyle, other environmental confounding factors, ethnicity characteristics, lifestyle, and socioeconomic status of the population are regarded as other limitations of the present study. The reason for the deviation from HWE is not clear in this population; it may have several causes such as small sample size, emigration, and immigration, or consanguineous marriages that are usual in this area of Iran.

## Conclusion

The present results emphasized the impact of NOS2 polymorphisms on T2D risk in the sample of Iranian population. Heterozygote genotypes of NOS2 rs2779248T/C and NOS2 rs1137933C/T gene polymorphisms were statistically associated with T2D as a risk factor and protective role, respectively. Further studies with larger sample sizes and different ethics are recommended.

## Ethical considerations

Ethical issues (Including plagiarism, informed consent, misconduct, data fabrication and/or falsification, double publication and/or submission, redundancy, etc.) have been completely observed by the authors.
